# Electrophysiological characterization of texture information slip-resistance dependent in the rat vibrissal nerve

**DOI:** 10.1186/1471-2202-12-32

**Published:** 2011-04-16

**Authors:** Fernando D Farfán, Ana L Albarracín, Carmelo J Felice

**Affiliations:** 1 Laboratorio de Medios e Interfases, Departamento de Bioingeniería, Universidad Nacional de Tucumán & Consejo Superior de Investigaciones Científicas y Técnicas, Tucumán, Argentina; 2 Laboratorio de Neurociencia, Facultad de Medicina, Universidad Nacional de Tucumán, Tucumán, Argentina

## Abstract

**Background:**

Studies in tactile discrimination agree that rats are able to learn a rough-smooth discrimination task by actively touching (whisking) objects with their vibrissae. In particular, we focus on recent evidence of how neurons at different levels of the sensory pathway carry information about tactile stimuli. Here, we analyzed the multifiber afferent discharge of one vibrissal nerve during active whisking. Vibrissae movements were induced by electrical stimulation of motor branches of the facial nerve. We used sandpapers of different grain size as roughness discrimination surfaces and we also consider the change of vibrissal slip-resistance as a way to improve tactile information acquisition. The amplitude of afferent activity was analyzed according to its Root Mean Square value (RMS). The comparisons among experimental situation were quantified by using the information theory.

**Results:**

We found that the change of the vibrissal slip-resistance is a way to improve the roughness discrimination of surfaces. As roughness increased, the RMS values also increased in almost all cases. In addition, we observed a better discrimination performance in the retraction phase (maximum amount of information).

**Conclusions:**

The evidence of amplitude changes due to roughness surfaces and slip-resistance levels allows to speculate that texture information is slip-resistance dependent at peripheral level.

## Background

Rodents as well as many mammals are characterized by the presence of vibrissae or whiskers located on both sides of the muzzle [1]. When rats acquire sensory information by actively moving their vibrissae, a neural code is manifested at different levels of the sensory system [2-4]. Research in the past 5 years has been extensive in the neural code at different stages of the whisker sensory pathway [5-8].

Arabzadeh et al, have demonstrated that a texture code would exist in peripheral afferent response of the vibrissal system and that spike rate would be the coding mechanism that underlies the textures discrimination in the primary somatosensory cortex [6]. In a previous study we have shown that it is possible to characterize the vibrissal sensory information by analyzing the multifiber discharge of a selected vibrissal nerve. We showed that the afferent activity amplitude (RMS values) could be related to the physical properties of the surfaces [9].

Here, we used a similar protocol to that detailed by Albarracín et al [9], but in this case we used different roughness surfaces (sandpapers). We have analyzed the afferent activity of a single vibrissa innervation (average activity) in order to relate the information obtained to the roughness of surfaces. For this purpose, we have calculated the amplitude of afferent activity by using Root Mean Square values (RMS) obtained during protraction and retraction phases. Here, we have also analyzed the effect of slip-resistance levels. In all cases, the differences among experimental conditions were quantified through the maximum amount of information by using Information Theory [10,11].

We found that the discrimination of lower roughness could be achieved at higher slip-resistance levels, whereas the low slip-resistance levels are more appropriate for higher roughness. We also showed that the experimental conditions are better accurately discriminated in retraction phase. Thus, we have demostrated that discrimination of rough surfaces would require a strategy based on the slip-resistance levels variation.

## Methods

### Procedures

Five Wistar adult male rats (300 g - 350 g), between 60 and 80 days, were used in our experiments. They were deeply anesthetized with urethane (1.5 g/Kg) and their temperature was maintained at 37° by a servo-controlled heating pad. Surgery consisted of exposing the infraorbital nerve as well as the two branches of the facial nerve (buccal and upper marginal mandibular) on the right side. The motor branches were dissected and transected proximally to avoid possible motor influences on the sensorial pathway. The stimulation electrodes were placed on their distal stumps to produce the contraction of the mystacial muscles. The deep vibrissal nerve innervating a vibrissal follicle (DELTA vibrissa) was identified with a high magnification of a dissecting microscope. The dissected nerve was also transected proximally and this action allowed eliminating discharges arriving from higher level of the sensorial pathway. To make sure that the nerve transection did not affect the functionality of the vibrissal nerve during our recording time, we test the falling of the nerve afferent activity throughout the time (data not shown). We conclude that the activity start decreasing 1 hour after the nerve section, so we never exceeded this space of time in our experiments. We used a bipolar electrode (insulated silver wire, 0.2 mm diameter) to record the multifiber afferent discharge of the vibrissal nerve selected. The recording electrodes as well as the nerves were immersed in a mineral oil bath during all recording.

All these procedures were carry out in accordance with the recommendations of the Guide for the Care and Use of Laboratory Animals (National Research Council, NRC). Depth of anesthesia was ascertained and controlled during the experiment by the lack of either withdrawal reflex to hindlimb pinching or blink reflex to a gentle stimulation of the cornea.

### Recording of the vibrissa electrical activity

In this study we have recorded the multifiber activity of the DELTA vibrissal nerve while the vibrissa was sweeping surfaces of different roughness. The experimental protocol used in this paper has been previously described in detail by Albarracín et al [9]. The procedures are briefly described below.

**Figure 1:**
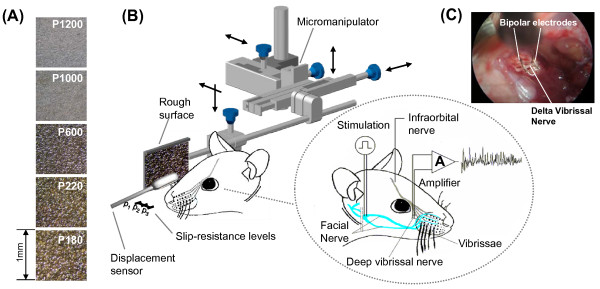
**Experimental set up**. (A) Surfaces Pictures. Photographs of the surfaces used in this paper. (B) It shows how the facial nerve must be stimulated for producing the artificial movement of the vibrissa, and the methodology used to obtain the recordings of the electrical activity in the deep vibrissal nerve (modified from [9]). The three slip-resistance levels were obtained by approaching the surfaces. At slip-resistance level 1 (p1), the vibrissa remains in contact with the surface without undergoing deformation. The slip-resistance levels 2 and 3 (p2 and p3) were obtained by using a micromanipulator, and bringing the surfaces closer to the vibrissa by 1 mm each time. (C) Recording site and placement of recording electrodes.

Vibrissa movements were induced by electrical stimulation of facial motor nerve (VII). Square-wave pulses (30 μs, 7 V supramaximal, 10 Hz) simulated vibrissal whisking at its natural frequency. A diagram of the experimental set up is shown in Figure 1B.

Nerve activity was recorded and digitized at 20 kHz during a 90 ms window following onset of each cycle of whisker movement (Digidata 1322A, Axon Instruments). Fifty whisker movement cycles were obtained for each surface, and an additional 50 cycles were recorded while whisker moved unobstructed in air (control).

Three levels of slip-resistance were presented for each swept surface. These levels were established by mounting the surface at different distances from the whisker base. A minimal slip-resistance level was presented by placing the surface at a maximal distance from the whisker base so that the tip just barely contacted the surface throughout the entire movement cycle (slip-resistance level 1). Increased slip-resistance levels were presented by moving the surface 1 and 2 mm closer to the whisker base (slip-resistance levels 2 and 3, respectively). This procedure to increase the slip-resistance is similar to those described by Albarracin et al [9].

Movements of the delta whisker were recorded simultaneously with nerve activity by using a custom-made photoresistive sensor (Figure 1B). The frequency response of the sensor was maximal in the range 0-100 Hz, enabling direct identification of the protraction and retraction phases of the movement cycle [12].

### Rough surfaces

The swept surfaces used in this paper were sandpapers of different grain size: P1200, P1000, P600, P220, and P180 (Figure 1A). We measured the surfaces roughness by using a Hommel Tester T1000 (Hommel Werke, http://www.hommel-etamic.de) and we used the Ra parameter (arithmetical deviation of the assessed profile) as a roughness estimation (International Standards BS.1134 and ISO 468). Ra values obtained were: 2.2, 2.9, 5.6, 8.9 and 9.2 μm, respectively.

### Digital processing and statistics

The RMS values and spectral estimation were previously used for the analysis of multifiber recordings [9]. Therefore, the theoretical details of both processing techniques can be found in the reference mentioned above. Here, the RMS values were obtained and analyzed in: (a) the whole whisker movement (protraction + retraction), (b) during the protraction and (c) during the retraction.

Statistical analysis was carried out with Kruskal Wallis ANOVA. The Kruskal Wallis test is a nonparametric alternative to the One-Way ANOVA. The test assesses the hypothesis that the different samples in the comparison were drawn from the same distribution or from distributions with the same median. Thus, the interpretation of the Kruskal-Wallis ANOVA is basically identical to the parametric One-Way ANOVA, except that it is based on ranks rather than on mean values.

The Dunn's method for multiple comparisons was used as post-hoc test, when ANOVA Repeat Measurement revealed a significant difference. These analyses were made with software SigmaStat http://www.systat.com/.

### Information theory

To determine the amount of information in a biological system it is necessary to have at least a pair of stimuli/responses situations. The stimulus may be a time series or simply belong to a class (for example, situation 1, situation 2, situation 3,..., situation N). The response depends on the characteristic of the signal that is being examined. Thus, they can be real values (RMS values, inter-spikes time, and others) or integer values (number of spikes).

The information conveyed by the neuronal response about the stimulus can be quantified by Shannon's mutual information formula [10], abbreviated hereafter as information:(1)

Where *P(s) *is the probability stimulus *s presentation, P(s|r) *is the posterior probability of *s *given the observation of response *r*, and *P(r) *is the probability of response *r *unconditional on the stimulus. The Information determines the maximum amount of knowledge (the upper bound of information) available to an observer who knows the posterior probabilities *P(s|r) *and uses them to read off the signals available in a single observation of a spike train [11].

For a better understanding of the procedure to determine the amount of information, we will consider a specific application in which the stimuli are given by the experimental situations (P1200, P1000, P600, P220 and P180). The responses will be real values, for example, the RMS values of the afferent activities. These real values will be in the range [*a, b*]. Where *a *and *b *are the maximum and minimum values of the response.

**Figure 2:**
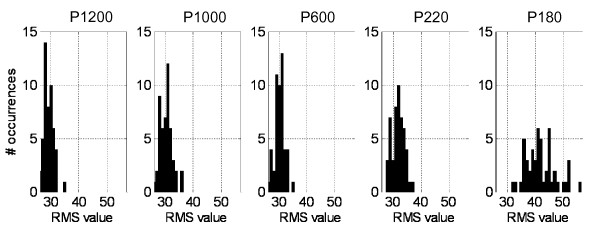
**Frequency diagrams of the RMS values**. These values were calculated from afferent activity registered during seep situations.

1. *First, the frequency diagrams (or histograms) determination*. For each experimental situation, the number of occurrences of the response is calculated for each bin. A graphical way to represent these diagrams is according to a histogram (Figure 2). The number of bins of the histograms will be calculated directly by the root square of the elements number (in this example, 50 RMS values).

2. *Determination of the joint probability distribution, P(s,r)*. The following Table 1 is made.

**Table 1:** Joint probability distribution

		*S*				
		
		P1200	P1000	...	P180	P(r)
**r**	***a***		.	.		
	**c**		.	.		
	.	.	.	.	.	.
	.	.	.	.	.	.
	.	.	.	.	.	.
	***b***		.	.		

**P(s)**			.	.		**Σ = 1**

The response and stimuli are r and s, and their marginal probabilities distributions are *P(r) *and *P(s)*, respectively

Each element of Table 1 is the joint probability value, *P(s,r)*. It is conceptually defined as follows: *P(P1200, r = a)*, it is the probability of obtaining a response *r = a *and that it corresponds to the sweep on P1200. This is:(2)

Where:

*#occurr_P1200,a _*is the occurrences number obtained for a value of r = a (first histogram of Figure 2).

*#total_occurr *is the total number of occurrences in all situations and all possible responses.

**Figure 3:**
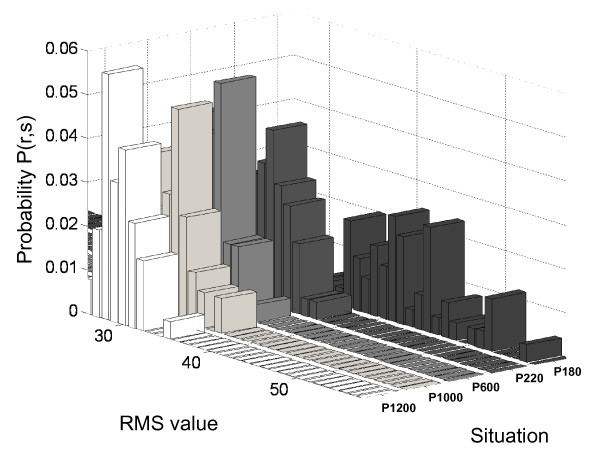
**Joint probability distribution**.

For frequency diagrams of Figure 2, the joint probability distribution is shown in Figure 3.

3. *Determination of the P(r) and P(s) probability distributions*. The probability of obtaining a response *r*, regardless of whether stimulus *s *did or not occur, is called the marginal probability, and it can be calculated by the sum of joint probabilities for a given response *r*. This is:(3)

Similarly, we obtain the probability function *P(s)*.(4)

4. *Determination of conditional probability distribution P(s|r)*. By definition of conditional probability, is given by:(5)

Then, conditional probabilities distributions for each stimulus are obtained from joint and marginal probabilities distributions. Thus, the following probabilities distributions are obtained: *P(P1200|r), P(P1000|r),..., P(P180|r)*.

**Figure 4:**
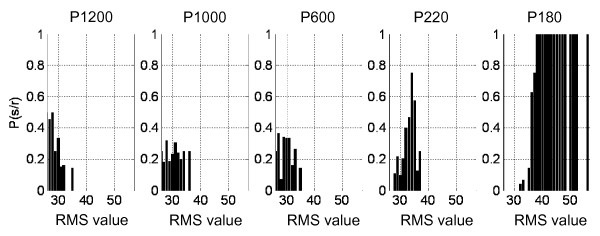
**Conditional probabilities distributions for each sweep situation**.

The conditional probability, *P(P1200|r)*, conceptually answer the question: Given a response *r*, what is the probability that this response has been produced by the P1200 stimulus?. Figure 4 shows the conditional probability distributions for sweep situations.

5. *Determination of the amount of information*. After obtaining all probability distributions, it is possible to obtain the mutual information using eq. 1.

In this paper, we quantified the information considering the RMS values as biological responses of the system. For the RMS values, the information was calculated for each slip-resistance level, as well as for each whisking phases (protraction and retraction).

## Results

**Figure 5:**
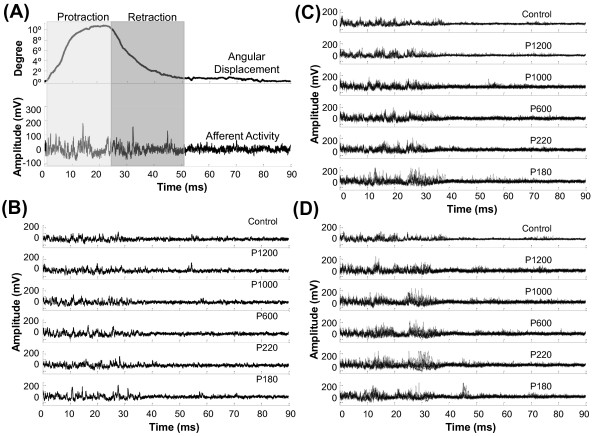
**Afferent discharges**. (A) Displacement and afferent activity recordings from DELTA vibrissa acquired during a sweep on sandpaper P600. The displacement recordings were acquired by using a custom-made photoresistive sensor. (B) Afferent discharges recorded when the vibrissa was sweeping: the air (control), sandpaper P1200, sandpaper P1000, sandpaper P600, sandpaper P200 and sandpaper P180. All recordings show a single vibrissa sweeping on each surface at slip-resistance level 1. (C) Ten afferent activity recordings obtained in different experimental conditions at slip-resistance level 1. (D) Idem to C at slip-resistance level 2.

The recordings obtained are the average electric activity of about 200 myelinated axons which supply innervation to the DELTA vibrissa. Figure 5B shows the afferent discharge recorded during different sweep situations (slip-resistance level 1). It is possible to distinguish amplitude changes in the signals in relation with the surface roughness. This feature is more appreciable in Figures 5C and 5D.

Whisker displacements and evoked afferent activity were simultaneously recorded for all cases. Figure 5A shows the afferent activity evoked by vibrissal sweeps in the air (control) and its corresponding displacement. Vibrissal movement recordings were acquired with a bandwidth of 0-100 Hz; thus, only slow movements were monitored. Therefore, displacement recordings were only used to identify the protraction and retraction phases.

**Figure 6:**
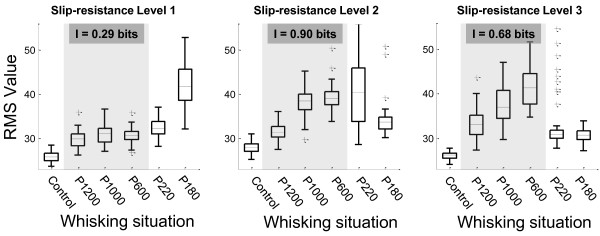
**Distribution of RMS values for each whisking situation**. Each experimental condition is described by 50 RMS values which were obtained from a whole-whisk cycle (protraction + retraction). In all cases the information values were obtained taking into account the first three sweep situations (shaded area).

We calculated one RMS value for each recording obtained during a vibrissa sweeping. Each experimental condition consists of 50 RMS values (one for each sweep). We first calculated the RMS values for the whole-whisk recordings (protraction + retraction). The distribution of RMS values for each sweep situation is shown in Figure 6.

Table 2 shows the statistical results of multiple comparisons among all sweep situations. These were obtained for the three slip-resistance levels. The controls were statistically different from the rest of situations, P < 0.05. These results are not shown in Table 2, nevertheless, they can be observed in Figure 6.

**Table 2:** Results of multiple comparisons applied to RMS values obtained from each sweep situation (Dunn method)

	Slip-resistance level 1			Slip-resistance level 2			Slip-resistance level 3		
**Comparison**	**Diff of Ranks**	**Q**	**P < 0.05**	**Diff of Ranks**	**Q**	**P < 0.05**	**Diff of Ranks**	**Q**	**P < 0.05**

**P1200 vs P1000**	32.670	1.883	No	119.330	6.878	Yes	54.930	3.166	Yes
**P1200 vs P600**	26.180	1.509	No	133.310	7.684	Yes	91.220	5.258	Yes
**P1200 vs P220**	70.440	4.060	Yes	125.270	7.220	Yes	26.810	1.545	No
**P1200 vs P180**	155.530	8.965	Yes	48.220	2.779	No	45.930	2.647	No
**P1000 vs P600**	6.490	0.374	No	13.980	0.806	No	56.290	3.092	Yes
**P1000 vs P220**	37.770	2.177	No	5.940	0.342	No	81.740	4.711	Yes
**P1000 vs P180**	122.860	7.082	Yes	71.110	4.099	Yes	100.860	5.813	Yes
**P600 vs P220**	44.260	2.551	No	8.040	0.463	No	118.030	6.803	Yes
**P600 vs P180**	129.350	7.456	Yes	85.090	4.905	Yes	137.150	7.905	Yes
**P220 vs P180**	85.090	4.905	Yes	77.050	4.441	Yes	19.120	1.102	No

The test computes statistic Q, the number of rank sums, and shows whether P < 0.05 or not, for the pair that are being compared. P is the probability that the null hypothesis may be rejected and, thus, it helps conclude that there are differences between treatments. Diff of ranks is the difference in the rank sum orders that are being compared. The rank sums are a measurement of the difference between two treatments.

The distribution of RMS values for the situations P1200, P1000 and P600, in slip-resistance level 1, are not significantly different among them (P > 0.05). However, P1200 and P1000 are significantly different when the slip-resistance level increased from 1 to 2. P1000 and P600 are not different statistically at slip-resistance level 2. Finally, P1200, P1000 and P600 situations were statistically different at slip-resistance level 3.

The significant differences are more remarkable at slip-resistance level 1 for higher roughness (P220 and P180). However, these differences are not significant at slip-resistance levels 2 and 3, probably because there are sudden shocks and stucks. Therefore, vibrissae movements are not repeatable, resulting in high dispersions of RMS value distributions (Figure 6 - P180 - slip-resistance Level 1). This effect is even more intensified when the slip-resistance level increases. At slip-resistance level 3, DELTA vibrissa sweeping on P220 and P180 results in low RMS values due to improper movement of DELTA vibrissa.

**Figure 7:**
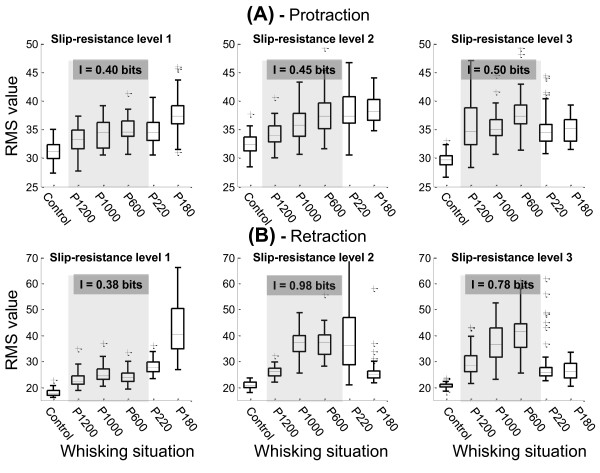
**Distribution of RMS values obtained for each whisking phase**. (A) RMS values obtained for the protraction phase. In all cases the information values were calculated taking into account the first three sweeps situations (shaded area). (B) RMS values obtained for the retraction phase.

Since our experimental protocol allows us to identify the protraction and retraction phases, we have obtained the RMS values in these two phases of the whisking cycle. The distribution of RMS values for each experimental condition and for the protraction phase is shown in Figure 7A. At slip-resistance level 1, the statistically different situations were: P1200 vs P600; P1200 vs P180; P1000 vs P180; P600 vs P180 and P220 vs P180. In all these cases, p-values were lower than 0.05. The increase of slip-resistance level (slip-resistance 2) produced statistical differences in P1200 vs P1000 and P1200 vs P220, which were not different at slip-resistance level 1. At slip-resistance level 3 two significant differences were produced: P1200 vs P600 and P600 vs P220. Table 3 shows the statistical results of these multiple comparisons.

**Table 3:** Results of multiple comparisons applied to distribution of RMS values

	PROTRACTION								
	
	Slip-resistance level 1			Slip-resistance level 2			Slip-resistance level 3		
**Comparison**	**Diff of Ranks**	**Q**	**P < 0.05**	**Diff of Ranks**	**Q**	**P < 0.05**	**Diff of Ranks**	**Q**	**P < 0.05**

**P1200 vs P1000**	40.800	2.352	No	51.750	2.983	Yes	10.160	0.586	No
**P1200 vs P600**	59.100	3.406	Yes	85.040	4.902	Yes	57.350	3.306	Yes
**P1200 vs P220**	48.220	2.779	No	99.850	5.755	Yes	10.120	0.583	No
**P1200 vs P180**	123.420	7.114	Yes	116.220	6.699	Yes	7.540	0.435	No
**P1000 vs P600**	18.300	1.055	No	33.290	1.919	No	47.190	2.720	No
**P1000 vs P220**	7.420	0.428	No	48.100	2.772	No	20.280	1.169	No
**P1000 vs P180**	82.620	4.762	Yes	64.470	3.716	Yes	2.620	0.151	No
**P600 vs P220**	10.880	0.627	No	14.810	0.854	No	67.470	3.889	Yes
**P600 vs P180**	64.320	3.707	Yes	31.180	1.797	No	49.810	2.871	No
**P220 vs P180**	75.200	4.334	Yes	16.370	0.944	No	17.660	1.018	No

	**RETRACTION**								
	
	**Slip-resistance level 1**			**Slip-resistance level 2**			**Slip-resistance level 3**		

**Comparison**	**Diff of Ranks**	**Q**	**P < 0.05**	**Diff of Ranks**	**Q**	**P < 0.05**	**Diff of Ranks**	**Q**	**P < 0.05**

**P1200 vs P1000**	40.370	2.327	No	108.260	6.240	Yes	63.280	3.647	Yes
**P1200 vs P600**	22.080	1.273	No	110.680	6.379	Yes	87.710	5.056	Yes
**P1200 vs P220**	96.750	5.577	Yes	97.380	5.613	Yes	24.840	1.432	No
**P1200 vs P180**	161.510	9.309	Yes	9.400	0.542	No	37.540	2.164	No
**P1000 vs P600**	18.290	1.054	No	2.420	0.139	No	24.430	1.408	No
**P1000 vs P220**	56.380	3.250	Yes	10.880	0.627	No	88.120	5.079	Yes
**P1000 vs P180**	121.140	6.982	Yes	117.660	6.782	Yes	100.820	5.811	Yes
**P600 vs P220**	74.670	4.304	Yes	13.300	0.767	No	112.550	6.487	Yes
**P600 vs P180**	139.430	8.037	Yes	120.080	6.921	Yes	125.250	7.219	Yes
**P220 vs P180**	64.760	3.733	Yes	106.780	6.155	Yes	12.700	0.732	No

These values are analyzed separately in protraction and retraction phases (Dunn's method). The parameters used in this analysis, such as Diff of Ranks, Q and P, are described in the legend of Table 2.

Figure 7B shows the distribution of RMS values obtained during the retraction phase. The sweep situations that were statistically significant were: P1200 vs P220; P1200 vs P180; P1000 vs P220; P1000 vs P180; P600 vs P220; P600 vs P180 and P220 vs P180 (P < 0.05). Therefore, seven out of ten comparisons were statistically different in retraction phase, whereas five out of ten were observed in protraction phase (slip-resistance level 1). Six comparisons were statistically different at slip-resistance level 2 and 3 (Table 3).

The information values obtained for the whole-whisking cycle (protraction + retraction) are shown in Figure 6. These values were calculated using three experimental situations (P1200, P1000 and P600). The P220 and P180 situations (rougher surfaces) were not included in the calculation due to the differences in the kinematic characteristics of vibrissal movement described above. The maximum amount of information was for slip-resistance level 2 (I = 0.9 bits). This information value is given mainly by the difference between situations P1200 vs P1000 and P1200 vs P600 (which are statistically different). Even though situations P1200, P1000 and P600 are statistically differents, the information value for slip-resistance level 3 is lower than for slip-resistance level 2. This is due to that the quantitative differences, in slip-resistance level 3, between P1200 vs P1000, P1200 vs P600 and P1000 vs P600 do not exceed the differences between P1200 vs P1000 and P1200 vs P600, in slip-resistance level 2. From this point of view, the information values provide a quantitative parameter of the differences between the sweep situations.

In the protraction phase, the information value was greater than in the whole-wisk in the slip-resistance level 1 (I = 0.40 bits). In this phase, the information values were 0.45 bits and 0.5 bits for slip-resistance levels 2 and 3, respectively (Figure 7A). In the retraction phase, the maximum amount of information was observed in slip-resistance level 2, I = 0.98 bits (Figure 7B).

These results show that surfaces discrimination would require a strategy based on the slip-resistance levels variation. Furthermore, the discrimination of lower roughness has been achieved at higher slip-resistance levels, whereas the low slip-resistance levels are more appropriate for higher roughness.

## Discussion

Texture encoding in the vibrissal system have being studied from the activity of trigeminal neurons and/or cortical neurons (barrel cortex neuron clusters). These responses usually consist of spikes or trains of spikes obtained from single or multi unit recordings [5,6]. Ideally, if the information from each constituent elements of the system is obtained, then the system would be fully described. However, this would be practically impossible due to the complexity of necessary instruments. The experimental procedures proposed here allow to observe (although in an approximate way) the system's global behaviour through a single recording (multifiber activity). In this paper the system's global behaviour refers to whole innervation of a vibrissal follicle. Contrary to the experimental protocols in which spikes are analyzed, this approach allows to makes inferences about activity of individual fibers.

Here we analyzed compound action potentials (CAP) recorded at the vibrissal nerve. The deep vibrissal nerve of the delta vibrissa consists of approximately 200 myelinated axons (data not shown, obtained from PhD thesis of Albarracín) [13]. These CAPs are the result of phase summation and cancellation of single fiber potentials with amplitudes that depend on fiber diameter, and the amplitude and shape of the CAP is determined by the distribution of fiber diameters. From the standpoint of digital processing these signals are considered to be stationary random process. Thus, for example, the temporal average would allow extracting the information from sensory nerves.

Generally, the electrophysiological signals obtained with techniques of single-unit and multi-unit recordings are statistically weak. It is easier to statistically analyze CAPs than single-unit recordings (because there is more information in CAP than single-unit recording) [14]. We have proposed the RMS method, because it presents temporal averaging. Another important aspect of this paper is the possibility to analyze the afferent activity in the preganglionic axons. The preganglionic recordings show the afferents activity without pre-processing.

The hypothesis of this research is that the changes of afferent signal amplitude (RMS value) would be related to different levels of mechanoreceptors activation [9], and these last ones would be roughness surfaces dependent [15]. As demonstrated by Wolfe et al, each roughness pattern evokes kinetic signatures characterized by slip-stick events [16]. These kinetic signatures would be encoded by spikes trains at both peripheral and central levels [6]. Thus, it is possible to speculate that changes in afferent signal amplitude may be related to temporal averages (spikes trains) and/or spatial averages (activity of many fibers) [17].

In this paper, the RMS value was employed in the same way as they were used by Albarracín et al [9]. However, a more detailed analysis was performed. The afferent activity was analyzed during the vibrissa protraction and retraction phases. In addition, we calculated the maximum amount of information that was possible to extract from RMS values.

We found that the lower roughness discrimination is achieved by using high slip-resistance levels (slip-resistance levels 2 and 3). On the other hand, higher roughness discrimination (P220 and P180) is achieved by using the lower slip-resistance (slip-resistance level 1). These results suggest that a behavioural strategy based on slip-resistance level changes could be used by rats in discrimination tasks. We have also observed that as roughness increased, the RMS values also increased in almost all cases. These observations are consistent with those obtained by others authors [9].

Information values were calculated considering only the first three sweep situations (P1200, P1000 and P600), because the kinetic characteristics of vibrissal sweep over surfaces of greater roughness, changes dramatically with the increase of slip-resistance level. In slip-resistance level 2, irregularities in vibrissal sweep (the vibrissa gets stuck in the surface) were observed, thus avoiding complete the normal sweeping (incomplete sweep). For this reason, high dispersions of RMS values were observed (sweep on P220, Figure 6, slip-resistance level 2). Under this slip-resistance level 2, and during the sweeps on P180, most of the sweeps were incomplete. By increasing the slip-resistance level to 3, the sweeps on P220 and P180, were incomplete sweeps. This particularity introduces unwanted factors in the evaluating of information when different slip-resistance levels are used. To avoid this, the first three sweep situations were used to calculate the information. Under these considerations, information values variations with slip-resistance levels were observed, being the maximum value for the slip-resistance level 2 (I = 0.9 bits). The information values obtained in protraction and retraction phases separately, shows that experimental situations are "better accurately" discriminated in retraction phase by using the slip-resistance level 2 (I = 0.98 bits).

Authors such as Arabzadeh et al. [6], Petersen et al. [18], Panzeri and Diamond [19] and others [20-23], have used the information theory to investigate exactly which features of ensemble neuronal activity report information about whisker stimuli. In other words, have investigated how sensory information is represented by neuronal populations in different stages of the sensory pathway. Unlike the researches mentioned above, here we used the Information Theory to demonstrate quantitatively the existence of texture information in the average afferent activity of a single vibrissa innervation and the dependence of this texture information with the slip-resistance.

### Methodological Considerations

#### Whisking in anesthetized rats

In this paper we induced artificial whisking in anesthetized rats and we recorded the afferent activity from vibrissal nerve during vibrissa sweeping on roughness surfaces. Although the principles of muscle-driven whisker movement and the basic patterns of movement trajectory are preserved in this kind of experimental protocols, the precise movements, mechanical interactions, and responses to touch may differ from those that occur during natural whisking [22-25]. Despite the inherent differences between natural and electrical whisking, the same kind of whisker motion was found in both experimental protocols [5,16,26]. Thus, experiments carried out in anesthetized animals, helps as the starting point for studies of multifiber activity in awake animals.

#### Facial Nerve Stimulation - Protocol

The vibrissa sweeping was produced by an electrical stimulation of the facial nerve. We used square-wave pulse to simulate the vibrissal whisking at its natural frequency (10 Hz). Whisking movements during artificial whisking may differ from those produced during natural whisking in some details. The vibrissa displacement angle observed in our experiments could differ from that of a behaving rat, as was previously described by Carvell and Simmons [27]. However our experimental protocol allows to record and to analyze the afferent activity without stimulus artifacts. The facial nerve stimulation with trains of pulses, as proposed by Szwed et al [25], it would contaminate the recording and could not be analyzed with current processing techniques.

## Conclusions

The evidence of amplitude changes due to roughness surfaces and slip-resistance levels allows to speculate that texture information is slip-resistance dependent at peripheral level, although the amplitude of the afferent activity is not biologically a plausible neural coding schemes of texture. Finally, we have demonstrated that it is possible to characterize the texture information by analyzing the afferent activity of a single vibrissa innervation.

## Competing interests

The authors declare that they have no competing interests.

## Authors' contributions

FDF and ALA designed and executed experimental protocols for acquisition of afferent signals, participated in the data interpretation and drafted the manuscript. ALA carried out the surgery procedures. FDF conceived, designed and implemented the algorithms. CJF assisted in digital processing and data interpretation. All authors read and approved the manuscript.
